# Organoid Technology in Precision Medicine for Head and Neck Cancer

**DOI:** 10.32604/or.2025.071296

**Published:** 2025-11-27

**Authors:** Boxuan Han, Shaokun Liu, Ridhima Das, Shiqian Liu, Yang Zhang

**Affiliations:** 1 Beijing Tongren Hospital, Department of Otorhinolaryngology Head and Neck Surgery, Capital Medical University, Beijing, 100730, China; 2 Key Laboratory of Otorhinolaryngology Head and Neck Surgery (Capital Medical University), Ministry of Education, Beijing, 100005, China; 3 Translational Radiation Oncology Research Laboratory, Department of Radiooncology and Radiotherapy, Charité—Universitätsmedizin Berlin (Corporate Member of Freie Universität Berlin and Humboldt Universität zu Berlin), Berlin, 10117, Germany; 4 Experimental Surgery, Department of Surgery, Charité—Universitätsmedizin Berlin (Campus Charité Mitte Campus Virchow-Klinikum, Affiliated with Freie Universität Berlin and Humboldt Universität zu Berlin), Berlin, 13353, Germany

**Keywords:** Organoid, head and neck squamous cell carcinoma, drug screening, tumor microenvironment, biomarker discovery

## Abstract

Organoid technology, characterized by high fidelity in mimicking the *in vivo* microenvironment, preservation of tumor heterogeneity, and capacity for high-throughput operations, has emerged as a critical tool in head and neck cancer research. To address clinical challenges in head and neck cancer management—including marked tumor heterogeneity, therapeutic resistance, and significant prognostic variability—this review focuses on four key translational applications of organoid technology: In mechanistic studies, organoid models provide a reliable platform for investigating tumorigenesis, progression, and drug resistance mechanisms. In personalized therapy, organoid-based drug sensitivity testing enables data-driven clinical decision-making. For biomarker discovery, organoids facilitate the identification of novel diagnostic markers and therapeutic targets. With ongoing improvements and standardization of organoid culture systems, this technology holds substantial promise for advancing precision medicine in head and neck cancer, bridging the gap between basic research and clinical practice.

## 1 Introduction

Head and neck cancer is one of the most prevalent malignancies globally, with over 900,000 new cases diagnosed annually and a five-year survival rate below 50%. Head and neck squamous cell carcinoma (HNSCC) accounts for the majority of these cases [1]. While combined modalities of surgery, radiotherapy, and chemotherapy have improved patient outcomes to some extent, clinical management remains challenged by several critical issues. First, chemoradiotherapy resistance is widespread: approximately 60% of patients with locally advanced disease (Stage III/IV) develop resistance to cisplatin-based concurrent chemoradiotherapy, leading to treatment failure [2]. Second, pronounced tumor heterogeneity exists, whereby cells from distinct regions of the same tumor exhibit marked variations in genomic, epigenetic, and microenvironmental characteristics, hindering precision therapy. Additionally, the lack of reliable individualized efficacy prediction models impedes clinicians from tailoring optimal treatment strategies for specific patients. These challenges underscore the urgent need for innovative research models and precision therapeutic approaches [3,4].

Traditional research models, such as cell lines and patient-derived xenografts (PDXs), have contributed to head and neck cancer research but possess inherent limitations. Cell lines lose primary tumor heterogeneity due to long-term *in vitro* passaging, while PDX models are time-consuming, costly, and fail to preserve the human immune microenvironment, and involve animal ethical restrictions due to reliance on immunodeficient animals for tumor transplantation [5]. Organoids, defined as three-dimensional (3D) *in vitro* culture systems derived from stem or progenitor cells (adult or pluripotent), exhibit self-organization capacity to recapitulate the structural architecture, cellular composition, and partial physiological functions of their corresponding *in vivo* organs [6]. For normal organoids across different tissues, they maintain tissue-specific differentiation characteristics: for example, normal salivary gland organoids form acinar-like and duct-like structures that can secrete amylase [7], while normal thyroid organoids retain follicular structures and iodine uptake ability [8]. In contrast, pathologic organoids (especially cancer organoids) are established from tumor tissues or cancer stem cells, and they preserve key malignant features of the original tumor, including histological heterogeneity, patient-specific genetic mutations, and responsiveness to therapeutic agents [5]. Notably, cancer organoids differ from normal organoids in their uncontrolled proliferation, disrupted tissue polarity, and ability to simulate tumor-stromal interactions within the tumor microenvironment (TME) [7]. Furthermore, organoids support high-throughput drug screening and real-time dynamic monitoring, providing a unique platform for studying tumor biology and developing personalized treatment strategies [9–11]. Fine-needle aspiration has been proposed as a minimally invasive method to establish organoids directly from patient tumors, preserving native tissue growth patterns and immune cell infiltration, thereby offering more physiologically relevant models for high-throughput drug screening and immunotherapy evaluation [12].

To date, various head and neck cancer organoid models have been successfully developed. Taking the establishment of organoids for three common types of head and neck tumors as examples, the establishment of these three head and neck cancer organoids all uses Matrigel as the matrix, contains Y-27632 (a Rho-Associated Protein Kinase [ROCK] inhibitor) in the culture medium, and requires cryopreservation and verification of consistency between the organoids and the original tumor. However, their key differences are as follows: In terms of dissociation, papillary thyroid carcinoma (PTC) uses collagenase type II combined with DNase I [13]; salivary gland cancer employs Liberase TM (research grade) and hyaluronidase [14]; HNSCC adopts enzymatic dissociation combined with mechanical dissociation [9]. Regarding the culture medium, PTC uses AdDMEM/F12 as the basal medium, supplemented with N-acetyl-L-cysteine, fibroblast growth factor-7/10 (FGF-7/10), SB202190 (a p38 Mitogen-Activated Protein Kinase [MAPK] inhibitor), and other relevant components; salivary gland cancer uses complete medium (with Y-27632 supplemented during passage); HNSCC uses Advanced DMEM as the basal medium, containing B27 cell supplement, FGF-b, prostaglandin E2 (PGE2), and Forskolin. In addition, the HNSCC medium requires the additional addition of 50% L-WRN conditioned medium (containing Wnt Family Member 3a [Wnt3a], R-Spondin 3 [RSPO3], and Noggin proteins), 10% R-Spondin 1 [RSPO1] conditioned medium, and 100 μg/mL Primocin (for contamination prevention). Scheemaeker et al. developed the first canine medullary thyroid carcinoma organoid model, with Single-nucleotide Polymorphism (SNP) genotyping validating its homology to the primary tumor [15]. Millen et al. established the largest head and neck cancer organoid biobank, comprising over 100 organoids from distinct patients—each linked to patients by tumor type (e.g., squamous cell carcinoma), TNM stage (e.g., T2N0M0), and location (e.g., oral cavity)—with a 71% recovery and expansion rate post-cryopreservation [16]. They also generated organoid models for two rare head and neck cancers and four salivary gland subtypes, validated via SNP genotyping (genetic homology), Whole-Exome Sequencing/Next-Generation Sequencing (WES/NGS) (molecular features), and retention of subtype-specific histology (e.g., salivary duct carcinoma’s cribriform structures). By comparing responses of 31 organoids to six targeted drugs, they observed marked inter-donor variability even with known actionable mutations, highlighting the potential of organoid cohorts in identifying drug-specific responders. Notably, radiosensitive organoids from 15 patients receiving adjuvant radiotherapy exhibited a significant correlation with longer recurrence-free survival [17]. Yang et al. demonstrated that papillary thyroid carcinoma organoids retain features of the original tumors, providing a novel preclinical model for research [13]. Mouse embryonic stem cell-derived salivary gland organoids, resembling embryonic salivary glands, serve as valuable tools for studying gland development and diseases [18]. Aizawa et al. established organoid models for multiple histological subtypes of human salivary gland cancers, including salivary duct carcinoma, mucoepidermoid carcinoma, and myoepithelial carcinoma; these models preserved original tumor histological features (e.g., cribriform structures in salivary duct carcinoma and cystic patterns in mucoepidermoid carcinoma) during passaging [14]. Tran et al. demonstrated that salivary gland-specific extracellular matrix-induced bone marrow-derived mesenchymal stem cells expressed various epithelial markers and exhibited morphology and ultrastructure similar to those of salivary glands. When implanted under the renal capsule of immunodeficient mice for 30 days, these aggregates formed functional salivary gland organoids expressing tissue-specific markers [19]. Zhang et al. successfully generated esophageal squamous cell carcinoma (ESCC) patient-derived xenografts and their corresponding organoids [20], while Takada et al. developed patient-derived adenoid cystic carcinoma organoids [21].

**Figure 1:**
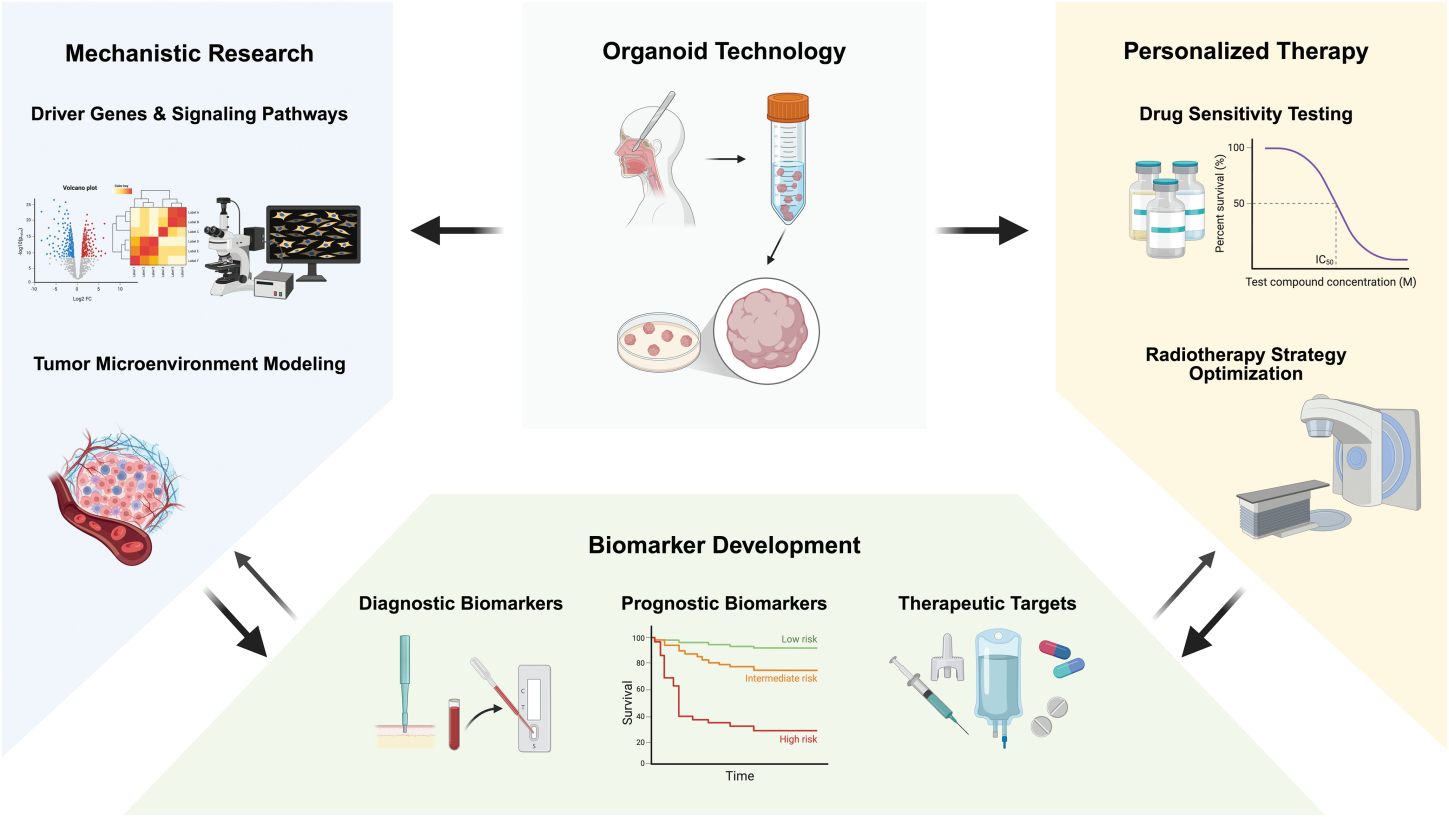
Schematic illustration of applications of organoids in translational medicine for head and neck cancer (This figure was created using BioRender, available at https://biorender.com/; developed by BioRender Inc., Toronto, ON, Canada)

This review systematically analyzes the translational value of organoid technology in head and neck cancer research, focusing on applications in mechanistic studies, personalized treatment decisions, and biomarker development. It also discusses current challenges in standardized culturing, genetic stability, and clinical translation, while envisioning integration of organoids with cutting-edge technologies such as single-cell sequencing and microfluidic chips. The overall framework of this review, encompassing its key focuses and aims, is summarized in Fig. 1; specifically, the framework centers on three core application directions of organoids and two key thematic discussions. The three application directions include: 1) the use of organoids in head and neck cancer pathogenesis research (covering functional validation of driver genes and tumor microenvironment simulation); 2) the enabling role of organoids in personalized therapy (including drug sensitivity testing and chemoradiotherapy sensitization/protection); 3) the promotional value of organoids in biomarker discovery (involving diagnostic biomarkers, prognostic biomarkers, and therapeutic targets). The two key thematic discussions refer to the existing challenges of organoid technology (standardized culturing, genetic stability, clinical translation) and future integration directions (combination with cutting-edge technologies like single-cell sequencing and microfluidic chips). Its aim is to provide theoretical foundations and practical guidance for advancing precision medicine paradigms in head and neck cancer.

## 2 Role of Organoids in Exploring the Pathogenesis of Head and Neck Tumors

### 2.1 Functional Validation of Driver Genes

Organoid models serve as an optimal research platform for functional validation of driver genes in head and neck cancers. By precisely simulating the tumor microenvironment and preserving the molecular features of primary tumors, organoid technology allows researchers to dissect the mechanisms of key oncogenes under near-physiological conditions. In recent years, multiple studies using this model have systematically validated the functions of various driver genes in head and neck carcinogenesis, yielding critical insights into tumor pathogenesis.

In esophageal cancer research, Klf5 has been shown to promote ESCC transformation by regulating Sox2 binding sites—specifically, it physically interacts with Sox2, guides Sox2 to newly opened chromatin regions and *de novo* super-enhancers (which drive the expression of oncogenes such as STAT3), stabilizes Sox2’s binding to these *de novo* sites, and forms a positive feedback loop with Sox2 (where Sox2 activates Klf5 transcription, and Klf5 in turn reinforces Sox2’s oncogenic binding activity). When combined with Trp53/Cdkn2a deletion, Sox2 can induce tumor formation from mouse esophageal organoids *in vivo* [22,23]. Ko et al. further employed Clustered Regularly Interspaced Short Palindromic Repeats (CRISPR)/Cas9 technology to demonstrate that dual knockout of Trp53 and Notch1 alone induces esophageal neoplasia, characterized by increased proliferation and loss of differentiation. Notably, simultaneous knockout of Trp53, Cdkn2a, and Notch1 yielded more aggressive tumors that recruited myeloid cells via Chemokine (C-C Motif) Ligand 2 (CCL2) secretion to form an immunosuppressive microenvironment, facilitating immune evasion [24]. Sun et al. elucidated the synergistic oncogenic interaction between CDKN2A-p16 deletion and KRASG12D activation, which significantly accelerates the progression from Barrett’s esophagus to dysplasia and activates multiple oncogenic pathways, including MAPK [25].

In salivary gland tumor research, Agaimy et al. identified a distinct KRAS codon 12 mutation subgroup in metaplastic Warthin tumors. Through systematic analysis of 22 cases, the researchers proposed reclassifying these tumors as “*de novo* proliferative Warthin tumors” with distinct neoplastic features, rather than traditional metaplastic lesions [26]. This discovery not only refined the molecular classification of the disease but also provided novel theoretical bases for clinical diagnosis and treatment.

Notable advances have also been achieved in thyroid cancer research. The Braf^V637E^ mutation has been confirmed to rapidly activate MAPK signaling, leading to cell dedifferentiation and disruption of follicular structures. This mutation induces downregulation of thyroid differentiation markers and enhanced proliferation, ultimately generating organoid models with characteristic thyroid cancer features [27]. Additionally, analysis of malignant teratoid tumors of the thyroid identified DICER1 “hotspot” mutations and TP53 missense variants, supporting the redefinition of these tumors as “thyroblastomas” [28]. Chen et al. further validated BRAF^V600E^’s role in 9 patient-derived PTC organoids (5 with BRAF^V600E^, PTC’s most common driver mutation), which stably cultured for >3 months and recapitulated parental tumors’ histology and mutations—confirming organoid BRAF mutations mirror clinical PTC characteristics. Clinically, this has translational value: for the 20% of PTC patients with recurrence/metastasis post-conventional therapy, BRAF inhibitors (vemurafenib, dabrafenib) alone showed mild efficacy in organoids (consistent with clinical observations), while combining them with MEK/RTK inhibitors or chemotherapeutics improved efficacy, providing preclinical evidence for guiding clinical targeted combinations [29].

These studies have systematically elucidated the functions of multiple key driver genes in head and neck carcinogenesis via organoid models. Across esophageal, salivary gland, and thyroid cancers, research teams have utilized organoid technology to reveal how driver gene mutations regulate tumor biology through specific signaling pathways. These findings not only deepen our understanding of head and neck cancer pathogenesis but also establish theoretical foundations for designing targeted therapeutic strategies. With ongoing advancements in organoid technology, its application potential in driver gene functional studies and personalized medicine is poised to expand further.

### 2.2 Tumor Microenvironment Modeling

Research on tumor microenvironment simulation has made groundbreaking advancements in head and neck cancer organoid models, offering novel insights into the complex interaction mechanisms between tumor cells and microenvironmental components. Through innovative organoid culture techniques, researchers can accurately recapitulate the dynamic characteristics of the tumor microenvironment, encompassing the regulatory roles of cancer-associated fibroblasts (CAFs), immune microenvironment features, signaling pathways, and other critical biological processes. These studies not only enhance the understanding of head and neck cancer pathogenesis but also establish a critical foundation for developing targeted therapeutic strategies targeting the tumor microenvironment.

As a key metabolite in tumor metabolism, lactate exerts intricate regulatory effects in the head and neck cancer microenvironment. Sharpe et al. obtained tumor tissues and CAFs from surgical resection specimens of esophageal adenocarcinoma (EAC) patients, subsequently culturing the tumor tissues into organoids. Through co-culturing CAFs and tumor organoids at a 2:1 ratio to form assembloids, RNA sequencing analysis revealed high expression of hypoxia markers in these constructs [30]. Zhao et al. validated the role of CAFs-lactate crosstalk in oral squamous cell carcinoma (OSCC): they isolated patient-derived OSCC organoid CD44^+^ cancer stem cells (via flow sorting) and co-cultured them with autologous CAFs in Matrigel, showing CAFs enhance CD44^+^ cell organoid-forming ability and upregulate stemness markers (CD44, OCT-4) via lactate—effects blocked by inhibiting lactate production/uptake. Key precautions include using patient-matched CAFs (to avoid heterogeneity) and ensuring CD44^+^ cell purity; clinically, this model recapitulates CAFs-driven OSCC recurrence-related stemness, supporting screening of lactate axis-targeted drugs [31]. Su et al. identified that lactate exerts a dual regulatory effect on EAC organoid growth: while reducing average organoid area and expression of proliferation marker Ki67, it enhances cellular heterogeneity. Mechanistic studies demonstrated that lactate inhibits glycolysis by altering the Nicotinamide Adenine Dinucleotide (Reduced Form, NADH)/Nicotinamide Adenine Dinucleotide (Oxidized Form, NAD^+^) redox state and may influence immune and metabolic pathways through regulation of ID1 and RSAD2 genes. Notably, pharmacological inhibition of lactate dehydrogenase (LDH) reversed lactate’s suppressive effect on EAC organoid growth [32]. These findings elucidate the intricate regulatory network of lactate metabolism in head and neck cancer progression, providing novel perspectives for metabolism-targeted therapies.

Organoid technology facilitates the elucidation of immune microenvironment characteristics in head and neck cancer. Liao et al. identified unique immune features in esophageal high-grade intraepithelial neoplasia via single-cell transcriptomics, including elevated Regulatory T Cells (Treg) cell counts and impaired CD8^+^ T cell function, which potentially facilitate immune evasion [33]. Baregamian et al. reported that co-culturing thyroid cancer organoids with T lymphocytes enhances immune cell cytokine secretion, while irradiation induces Nrf2-mediated antioxidant responses [34]. Shin et al. employed organoid technology to validate trogocytosis—a process where cancer cells acquire immune cell surface markers (e.g., CD4) from tumor-infiltrating lymphocytes—in PDX models of head and neck cancer [35].

Organoid technology deciphers the mechanisms by which signaling pathways within the TME regulate cancer progression. Murakami et al. compared metabolic profiles of tongue cancer cells across culture conditions and found that 3D organoid cultures better recapitulate *in vivo* metabolic characteristics, including enhanced glycolysis and Tricarboxylic Acid Cycle (TCA) cycle activity [36]. Zhao et al. characterized the Nicotinamide N-Methyltransferase (NNMT)-Lysyl Oxidase (LOX)-Focal Adhesion Kinase (FAK) regulatory axis in the OSCC microenvironment via organoid models. NNMT was significantly upregulated in OSCC-associated CAFs, with fibroblast-attached organoids partially recapitulating this phenotype. NNMT inhibition reduced CAF activation and organoid regenerative capacity, whereas its overexpression promoted regeneration. Mechanistically, NNMT facilitated tumor initiation and growth by increasing type I collagen deposition and sustaining FAK signaling to preserve cancer stemness. Epigenetically, NNMT enhanced LOX transcription through reduced histone methylation, further driving collagen deposition in the TME [37]. Zhang et al. established an organoid model demonstrating that acidic bile salts induce epithelial-mesenchymal transition (EMT), potentially contributing to esophageal intestinal metaplasia [38]. Anand et al. co-cultured Barrett’s esophagus organoids with fibroblasts, showing that Nuclear Factor Kappa-Light-Chain-Enhancer of Activated B Cells (NF-κB) β CCL5/CCL20 levels and prevented dysplasia development [39]. Diaz et al. demonstrated that Wnt/β-catenin inhibitor pyrvinium markedly suppressed anaplastic thyroid cancer (ATC) cell lines and patient-derived ATC organoids, with enhanced efficacy in BRAF^V600E^-mutated organoids when used in combination with standard therapy [40]. Czerwonka et al. validated the anticancer activity of Notch modulator RIN-1 in HNSCC organoids [41].

In summary, organoids are invaluable for validating driver genes and tumor-microenvironment interactions in head and neck cancer. They also facilitate deciphering chemotherapy resistance mechanisms. These roles of organoids in exploring the pathogenesis of head and neck tumors are summarized in Table 1. Su et al. employed HNSCC patient-derived organoids to demonstrate that chemotherapy-resistant CAFs promote drug resistance via Transforming Growth Factor Alpha (TGFα)-Epidermal Growth Factor Receptor (EGFR) paracrine signaling, thereby activating Phosphatidylinositol 3-Kinase (PI3K)/Protein Kinase B (AKT)/p65 to suppress p53/caspase-3-mediated apoptosis. Cetuximab restored chemosensitivity by blocking TGFα in CAFs [42]. Jiang et al. identified carboxylesterase 1 (CES1) as a mediator of cisplatin resistance in HNSCC organoids [43]. Organoid models also enable assessment of environmental carcinogens: Yoo et al. exposed thyroid cancer organoids to perfluoroalkyl carboxylates (PFCAs, 10 μM) and observed decreased Thyroid-Stimulating Hormone Receptor (TSHR) expression, elevated Thyroglobulin (Tg) levels, cytoplasmic relocalization of E-cadherin, upregulation of vimentin, and increased Ki-67+ cells—findings suggesting PFCAs promote proliferation and metastasis via EMT [44].

**Table 1:** Role of organoids in exploring the pathogenesis of head and neck tumors

Category	Tumor type	Key factors/ genes/mutations	Signaling pathway	Biological effects	References
Functional validation of driver genes	EC	Klf5, Sox2, Trp53, Cdkn2a, Notch1	Sox2/Trp53/Notch1	Cell transformation, proliferation, immune	[22–24]
	EC	CDKN2A-p16 deletion, KRAS^G12D^ activation	KRAS/MAPK	Dysplasia progression	[25]
	SGT	KRAS codon 12 mutation	KRAS pathway	Warthin tumor	[26]
	TC	Braf^V637E^ mutation	MAPK signaling	Cell dedifferentiation, hyperproliferation	[27]
	TC	DICER1 “hotspot” mutations, TP53 variants	RAS/MAPK pathway	Thyroblastoma redefinition	[28]
Tumor microenvironment modeling	EAC, OSCC	Lactate, CAFs	ID1/RSAD2 redox	Tumor growth regulation, tumor promotion	[30–32]
	EHGIN	Treg cells, CD8^+^ T cells	Treg/immune-checkpoint axis	Immune evasion	[33]
	TC	T lymphocytes	Nrf2 antioxidant pathway	Immune activation, antioxidation	[34]
	HNC	Tumor-infiltrating lymphocytes	immune tolerance	Trogocytosis/immune signaling	[35]
	TgCa	Glycolysis, TCA cycle	metabolic modeling	TCA cycle/glycolysis pathway	[36]
	OSCC	NNMT-LOX-FAK axis	LOX/FAK signaling	CAF activation, stemness maintenance	[37]
	EC	Acidic bile salts	EMT signaling	Intestinal metaplasia	[38]
	BE	NF-κB pathway	NF-κB pathway	Dysplasia prevention	[39]
	ATC	Wnt/β-catenin pathway	Wnt/β-catenin pathway	Tumor progression inhibition	[40]
	HNSCC	Notch pathway	Notch signaling	Tumor progression inhibition	[41]
	HNSCC	Chemoresistant CAFs, TGFα-EGFR	PI3K/AKT/p65 pathway	Drug resistance, cetuximab sensitivity restoration	[42]
	HNSCC	CES1	CES1/cisplatin resistance axis	Cisplatin resistance	[43]
	TC	PFCAs	EMT pathway	Cell proliferation, metastasis	[44]

Note: SGT: Salivary Gland Tumor; KRAS: Kirsten Rat Sarcoma Viral Oncogene Homolog; RAS: Rat Sarcoma Viral Oncogene Homolog Family; MAPK: Mitogen-Activated Protein Kinase; EAC: Esophageal Adenocarcinoma; OSCC: Oral Squamous Cell Carcinoma; CAFs: Cancer-Associated Fibroblasts; EHGIN: Esophageal High-Grade Intraepithelial Neoplasia; Treg: Regulatory T Cells; TC: Thyroid Cancer; HNC: Head and Neck Cancer; TgCa: Tongue Cancer; TCA: Tricarboxylic Acid Cycle; NNMT: Nicotinamide N-Methyltransferase; LOX: Lysyl Oxidase; FAK: Focal Adhesion Kinase; EC: Esophageal Cancer/Epidermoid Carcinoma (subtype); BE: Barrett’s Esophagus; NF-κB: Nuclear Factor Kappa-Light-Chain-Enhancer of Activated B Cells; ATC: Anaplastic Thyroid Cancer; HNSCC: Head and Neck Squamous Cell Carcinoma; Wnt: Wingless/Integrated (signaling pathway); TGFα: Transforming Growth Factor Alpha; EGFR: Epidermal Growth Factor Receptor; PI3K: Phosphatidylinositol 3-Kinase; AKT: Protein Kinase B; CES1: Carboxylesterase 1; PFCA: Perfluoroalkyl Carboxylates; EMT: Epithelial-Mesenchymal Transition.

## 3 Role of Organoids in Establishing Therapeutic Platforms for Head and Neck Cancer

### 3.1 Drug Sensitivity Testing

Organoid technology has demonstrated significant clinical utility in drug sensitivity testing for head and neck cancers, providing a novel research tool for developing personalized treatment strategies [45]. Multiple studies have demonstrated that head and neck cancer-derived organoids can highly recapitulate the histological characteristics and molecular profiles of primary tumors while retaining differential sensitivity to chemotherapeutic agents [45,46]. For example, head and neck cancer organoids not only serve as preclinical models for predicting chemotherapy responses but also exhibit significant correlations between their drug sensitivity parameters (e.g., Half-Maximal Inhibitory Concentration [IC_50_], Half-Maximal Effective Concentration [EC_50_], and Area Under the Curve [AUC]) and clinical outcomes. This finding has been validated in studies employing patient-derived organoids (PDOs) of EAC treated with cisplatin and paclitaxel [46].

In the field of targeted therapy, organoid models exhibit unique heterogeneity in drug response patterns [45,47]. Although the PIK3CA inhibitor alpelisib has been approved for the treatment of PIK3CA-mutated breast cancer [47], studies on head and neck cancer organoids demonstrate that its efficacy does not significantly correlate with PIK3CA mutation status, suggesting that tumor type may influence targeted drug sensitivity patterns [45]. Notably, organoids with specific genetic alterations, such as CDKN2A deletion, exhibit marked sensitivity to Protein Arginine Methyltransferase 5 (PRMT5) inhibitors [16], highlighting the value of organoids in identifying biomarker-drug associations. Significant progress has also been made in research on different tumor subtypes. For example, patient-derived organoid models of salivary gland carcinoma not only retain 97.6% of Catalogue Of Somatic Mutations In Cancer (COSMIC)-annotated variants and critical gene rearrangements but also accurately reflect subtype-specific differential responses to agents such as erlotinib in adenoid cystic carcinoma [48].

Organoid research in thyroid cancer has further extended the utility of this technology in precision medicine [49]. Papillary thyroid carcinoma organoids harboring the BRAF^V600E^ mutation not only exhibit long-term culture stability but also effectively predict the superior efficacy of BRAF/MEK inhibitor dual therapy, whereas wild-type organoids display the expected resistance profile [49]. Similarly, studies of esophageal cancer organoids have confirmed that their response patterns to cisplatin, paclitaxel, and proton beam therapy closely correlate with patient clinical outcomes. RNA sequencing has revealed gene expression heterogeneity, offering novel insights into treatment resistance mechanisms [50]. Driehuis et al. validated the utility of 31 patient-derived HNSCC organoids—recapitulating genetic and molecular traits of primary tumors (e.g., 69% TP53 mutation rate)—in drug sensitivity testing for HNSCC. Notably, in 7 patients receiving radiotherapy, organoid radiosensitivity (quantified by AUC) correlated with clinical outcomes: organoids from 3 patients with disease recurrence were among the most resistant, while radiosensitive organoids matched those from patients with sustained remission. Key strategies included covering standard HNSCC therapies (cisplatin, carboplatin, cetuximab, radiotherapy) with a significant correlation between cisplatin and carboplatin sensitivity (r = 0.64, *p* < 0.05), screening non-standard targeted agents (e.g., alpelisib, vemurafenib) to identify subtype-specific responses (e.g., BRAF^V600E^-mutant organoid T9 exhibited higher sensitivity to vemurafenib), and evaluating chemoradiation combinations (cisplatin enhanced radiotherapy efficacy in 6/10 organoid lines). For clinical translation, an HNSCC organoid biobank was established (accessible via www.hub4organoids.eu), and the observational trial ONCODE-P2018-0003 was initiated to link organoid drug/radiotherapy responses to patient outcomes, aiming to standardize the “biopsy-organoid testing-therapy selection” workflow for HNSCC [51]. Notably, a longitudinal study of salivary gland secretory carcinoma found that organoids generated pre- and post-treatment can dynamically capture acquired resistance mutations. However, discrepancies between predicted sensitivity to repotrectinib and clinical outcomes highlight the necessity of optimizing culture systems to enhance predictive accuracy [52].

Overall, organoid models exhibit substantial potential and unique technical advantages in drug testing for head and neck cancers [16,45,46,48]. From predicting chemotherapy sensitivity and screening targeted drugs to optimizing combination therapies, organoid technology provides reliable experimental evidence for clinical decision-making [49,50]. However, current studies also highlight technical challenges, including inconsistencies in predictive accuracy and variable culture success rates [52]. Future efforts should focus on optimizing microenvironmental simulation and establishing standardized protocols to enhance translational value. With the accumulation of additional clinical validation data, organoid technology is poised to become a critical decision-supportive tool for personalized treatment in head and neck cancers [45,48].

### 3.2 Radiosensitization and Radioprotection

Organoid models exhibit unique value in head and neck cancer radiotherapy research, serving as a critical platform for investigating radiosensitization mechanisms and developing radioprotection strategies [53]. Studies have shown that HNSCC PDOs not only retain histopathological and genomic characteristics of primary tumors but also display variations in radiation sensitivity that correlate with patient prognosis. The overall culture success rate of 35% increases to 77% in samples with ≥30% tumor cellularity, supporting the feasibility of personalized radiotherapy planning [53]. In radiosensitization research, cisplatin and carboplatin significantly augment radiotherapy efficacy, whereas cetuximab exhibits radioprotective properties—and these drug-radiation interactions are precisely quantified in organoid models [16]. Notably, AMP-Activated Protein Kinase (AMPK) pathway activation diminishes radiosensitivity in EAC cells and organoids, and pharmacological inhibition of this pathway markedly enhances radiation-induced cytotoxicity, thereby identifying a molecular target for novel radiosensitizers [54].

Salivary gland radioprotection studies utilizing organoid models have yielded groundbreaking findings [10,55,56]. Compared to traditional Two-Dimensional (2D) cultures, 3D organoid systems better recapitulate the complex architecture of salivary glands, preserving cell-cell interactions and extracellular matrix synthesis, thereby providing a physiologically relevant experimental platform for investigating radiation-induced damage [55,57,58]. Radiation induces mitochondrial dysfunction in salivary gland stem/progenitor cells, characterized by reduced membrane potential, Reactive Oxygen Species (ROS) accumulation, and aberrant mitochondrial networks, while pharmacological activation of mitophagy markedly enhances organoid self-renewal capacity [10]. Senescent cell clearance strategies exhibit therapeutic potential: ganciclovir and ABT263 administration effectively eliminate radiation-induced senescent cells, restoring both organoid formation efficiency and secretory function [56]. Mesenchymal stem cell-derived hepatocyte growth factor (HGF) promotes salivary gland stem cell proliferation via the mesenchymal-epithelial Transition Factor (cMET) receptor, mitigating premature senescence caused by low-dose radiation (3–7 Gy), though its reparative effects are limited in high-dose radiation-induced injury [59]. Murine parotid organoid studies further confirm the multipotent differentiation potential of acinar stem cells, whose radiosensitivity parallels that of submandibular glands, with Wingless/Integrated (Wnt) signaling exerting a key regulatory role in post-radiation tissue regeneration [60].

Organoid models offer novel insights into refining radiotherapy approaches [61–63]. Canine follicular thyroid cancer organoids maintain expression of iodine uptake-related proteins (e.g., thyroglobulin and sodium-iodide symporter [NIS]), providing an ideal *in vitro* platform for optimizing radioactive iodine therapy [61]. Nasopharyngeal carcinoma organoid studies demonstrate subtype-specific differences in chemoradiation sensitivity: the epidermoid carcinoma (EC) subtype exhibits a synergistic response to EGFR inhibitors in combination with radiotherapy, while squamous cell carcinoma (SC) and mixed squamous cell and epidermoid carcinoma (MSEC) subtypes show greater sensitivity to microtubule inhibitors—findings that inform precision combination therapy [62]. Mouse esophageal organoid experiments reveal that high-Linear Energy Transfer (LET) iron ion radiation induces more severe DNA damage and differentiation abnormalities than low-LET cesium radiation, with even low doses inducing sustained stress responses, which inform clinical radioprotection strategies [63].

Organoid technology has been revolutionizing head and neck cancer radiotherapy research [16,53,54]. From elucidating radiosensitivity mechanisms to validating combination therapies, and from exploring radioprotection strategies to optimizing dose parameters, organoid models demonstrate multifaceted applications. Current studies not only confirm their reliability in predicting radiotherapy responses [53,62] but also identify potential therapeutic targets, including the AMPK pathway and mitophagy [10,54]. However, challenges remain, such as variable culture success rates [53] and limited repair capacity for high-dose radiation-induced damage [59]. Future research should prioritize culture system optimization, microenvironment integration, and large-scale clinical validation to translate organoid technology into clinically actionable tools for enhancing radiotherapy outcomes [55,60]. These roles of organoids in establishing therapeutic platforms for head and neck cancer are summarized in Table 2.

**Table 2:** Role of organoids in establishing therapeutic platforms for head and neck cancer

Category	Tumor type	Key factors/drugs/mutations	Signaling pathway	Biological effects	References
Drug sensitivity testing	HNC (incl. EAC)	Cisplatin, paclitaxel	DNA damage response pathway	Chemotherapy sensitivity prediction	[45,46]
	HNC	PIK3CA inhibitor alpelisib	PI3K-AKT pathway	PI3K inhibitor sensitivity	[45,47]
	HNC	CDKN2A deletion, PRMT5 inhibitors	PRMT5-CDKN2A epigenetic axis	Biomarker-drug correlation	[16]
	SGC (incl. ACC)	Erlotinib	EGFR signaling	EGFR-targeted subtype response	[48]
	PTC	BRAF^V600E^ mutation, BRAF/MEK inhibitor combination	MAPK pathway	BRAF/MEK-targeted therapy guidance	[49]
	EC	Cisplatin, paclitaxel, proton beam therapy	DNA damage response pathway	Therapy response prediction	[50]
	SSC	Acquired resistance mutations, repotrectinib	ETV6-NTRK3 pathway	Resistance profiling	[52]
Radiosensitization and Radioprotection	HNSCC	Cisplatin, carboplatin, cetuximab, radiotherapy	EGFR pathway, radiosensitivity axis	Radiotherapy stratification	[16,53]
	EAC	AMPK pathway, AMPK inhibitors	AMPK signaling pathway	Radiosensitization enhancement	[54]
	Salivary gland	Mitophagy modulators, ganciclovir, ABT263	Mitochondrial damage signaling	Radioprotection via mitochondrial recovery	[10,56]
	Salivary gland	Mesenchymal stem cell-derived HGF, cMET receptor	HGF/MET signaling	Radioprotection via HGF signaling	[59]
	Murine parotid gland	Wnt signaling pathway	Wnt signaling	Regeneration after irradiation	[60]
	Canine FTC	Thyroglobulin, NIS	NIS-mediated iodine uptake pathway	Radioiodine therapy optimization	[61]
	NPC	EGFR inhibitors, microtubule inhibitors, chemoradiation	EGFR signaling and microtubule disruption	Chemoradiation subtype guidance	[62]
	(Model research)	High-LET iron ion radiation, low-LET cesium radiation	Radiation stress signaling	Radiation damage modeling	[63]

Note: HNC: Head and Neck Cancer; EAC: Esophageal Adenocarcinoma; SGC: Salivary Gland Carcinoma; ACC: Adenoid Cystic Carcinoma; PTC: Papillary Thyroid Carcinoma; EC: Esophageal Cancer; SSC: Salivary Gland Secretory Carcinoma; HNSCC: Head and Neck Squamous Cell Carcinoma; MAPK: Mitogen-Activated Protein Kinase; cMET: Mesenchymal-Epithelial Transition Factor; Wnt: Wingless/Integrated (signaling pathway); FTC: Follicular Thyroid Cancer; NIS: Sodium-Iodide Symporter; NPC: Nasopharyngeal Carcinoma; LET: Linear Energy Transfer.

## 4 Role of Organoids in the Discovery of Biomarkers for Head and Neck Cancer

Organoid models have emerged as a pivotal tool for biomarker development in head and neck cancers, providing a novel platform for identifying diagnostic markers, prognostic indicators, and therapeutic targets [64,65]. Studies demonstrate that head and neck cancer organoids retain high fidelity to the molecular features and heterogeneity of primary tumors, thereby enhancing the clinical relevance of organoid-based biomarker screening [64,66]. For instance, through the establishment of a biobank comprising 45 salivary gland tumor organoids, researchers successfully identified subtype-specific biomarkers such as protein tyrosine phosphatase 4A1 (PTP4A1), offering new diagnostic criteria for mucoepidermoid carcinoma [64]. Similarly, single-cell RNA sequencing of adenoid cystic carcinoma organoids revealed PRMT5 upregulation correlated with MYB/MYC genes—a finding that not only uncovered novel biomarkers but also validated PRMT5 inhibitors as a viable therapeutic strategy currently in phase I clinical trials [67].

Organoid models demonstrate distinct advantages in prognostic biomarker development [68,69]. Research on HNSCC organoids demonstrated that collective invasion patterns correlate strongly with YAP signaling activation, facilitating the establishment of a gene signature predictive of poor patient outcomes [68]. Human Papillomavirus (HPV)-negative HNSCC organoids further confirmed that high p-mTOR/p-ERK expression correlates with worse survival and predicts therapeutic resistance to mTOR inhibitor everolimus [69]. EAC studies integrated organoid models with long-read sequencing to resolve complex genomic amplification events, revealing potential early-diagnostic biomarkers [70]. Notably, cancer-fibroblast interaction-based organoid models accurately predict immunotherapy responses in HNSCC patients, with high interaction gene scores correlating strongly with immunosuppressive microenvironments and therapeutic resistance [65].

Organoids serve as a critical tool in therapeutic target discovery [71–73]. CRISPR-mediated SIRT7 knockout in HNSCC organoids validated its oncogenic role while demonstrating enhanced 5-fluorouracil (5-FU) chemosensitivity following deletion [73]. RNA Interference (RNAi) screening coupled with organoid models identified Fibroblast Growth Factor Receptor 4 (FGFR4) as a key target in HPV-negative HNSCC, inhibition of which reverses EMT—specifically, in sensitive cells, it leads to significant downregulation of mesenchymal markers such as Vimentin and upregulation of epithelial markers—and improves radiosensitivity [71]. In immunotherapy, ESCC organoids validated CD276 as an ideal Chimeric Antigen Receptor-Natural Killer (CAR-NK) target, with patient-derived organoid results showing perfect alignment with humanized mouse models [72]. Adenoid cystic carcinoma organoid studies revealed the superior efficacy of proteolysis-targeting chimera dBET6 (targeting Bromodomain-Containing Protein 4 [BRD4]) when compared to conventional inhibitor JQ1, particularly in myoepithelial-rich tumors [66].

Organoid technology is ushering in a new era in head and neck cancer biomarker research [74–76]. From circulating small extracellular vesicle miRNA classifiers to Bone morphogenetic protein (BMP) 2/4 pathway validation in Barrett’s esophagus carcinogenesis [74,75], to molecular dissection of racial disparities in EAC [76], organoids provide a robust translational platform. These roles of organoids in the discovery of biomarkers for head and neck cancer are summarized in Table 3. Current studies not only confirm their efficacy in biomarker discovery [64,67] but also highlight their clinical decision-guiding potential [65,69]. However, challenges such as standardization and heterogeneity preservation persist [68]. Future efforts should integrate multi-omics data, optimize co-culture systems, and perform prospective clinical trials to accelerate clinical translation [70,72]. With technological refinement, organoid models are poised to become the cornerstone of biomarker development for precision medicine in head and neck cancers [66,71].

**Table 3:** Role of organoids in the discovery of biomarkers for head and neck cancer

Category	Tumor type	Key factors/genes/biomarkers	Core mechanisms	Biological effects	References
Diagnostic biomarkers	SGT (incl. MEC)	PTP4A1	PTP4A1 subtype signaling	Diagnostic biomarker identification	[64]
	ACC	PRMT5, MYB/MYC	PRMT5-MYB/MYC axis	Novel biomarker validation	[67]
	EAC	Complex genomic amplifications	Genomic amplification pathways	Early diagnostic biomarker discovery	[70]
Prognostic biomarkers	HNSCC	YAP signaling	YAP signaling pathway	Prognostic model construction	[68]
	HPV-HNSCC	p-mTOR/p-ERK	mTOR/ERK signaling pathway	Resistance biomarker identification	[69]
	HNSCC	Cancer-fibroblast interaction scores	CAF-immune interaction signaling	Immune response prediction	[65]
Therapeutic targets	HNSCC	SIRT7	SIRT7 signaling axis	Chemo-sensitivity biomarker validation	[73]
	HPV-HNSCC	FGFR4	FGFR4/EMT signaling pathway	Radio-combination target identification	[71]
	ESCC	CD276	CD276 immune checkpoint signaling	CAR-NK immunotherapy targeting	[72]
	ACC	BRD4, dBET6 (PROTAC)	BRD4 epigenetic regulation	Epigenetic therapy development	[66]
Other biomarker applications	HNT	Circulating exosomal miRNA classifiers	miRNA signaling axis	Non-invasive diagnosis development	[74]
	BE	BMP2/4 pathway	BMP signaling pathway	Carcinogenesis marker discovery	[75]
	EAC	Racial disparity molecules	Race-associated regulatory pathways	Personalized biomarker discovery	[76]

Note: SGT: Salivary Gland Tumor; MEC: Mucoepidermoid Carcinoma; ACC: Adenoid Cystic Carcinoma; PTP4A1: Protein Tyrosine Phosphatase 4A1; PRMT5: Protein Arginine Methyltransferase 5; EAC: Esophageal Adenocarcinoma; HNSCC: Head and Neck Squamous Cell Carcinoma; YAP: Yes-Associated Protein; HPV: Human Papillomavirus; CAF: Cancer-Associated Fibroblast; EMT: Epithelial-Mesenchymal Transition; CAR-NK: Chimeric Antigen Receptor-Natural Killer; PROTAC: Proteolysis-Targeting Chimera; HNT: Head and Neck Tumor; BE: Barrett’s Esophagus; BMP: Bone Morphogenetic Protein.

## 5 Discussion

The application of organoid technology in head and neck cancer research marks a groundbreaking advancement in tumor modeling. Its unique advantage resides in its capacity to faithfully preserve cancer stem cells and differentiated cell lineages while accurately recapitulating dynamic tumor–stromal interactions within the TME [45]. For instance, via co-culture systems integrating CAFs and tumor-associated macrophages (TAMs), organoids successfully recapitulate the features of an immunosuppressive TME, thereby providing a powerful tool for investigating tumor-stroma interactions and therapy resistance mechanisms [31]. Additionally, patient-derived organoids (PDOs) exhibit high concordance with clinical therapeutic outcomes in drug sensitivity assays, offering reliable evidence for personalized treatment decisions [22,77]. Such technological advancements have not only enhanced our understanding of head and neck cancer pathogenesis but also significantly accelerated the clinical translation of precision medicine [55].

Despite its potential, the widespread application of organoid technology remains limited by several unresolved challenges. A key limitation lies in the fact that conventional PDO systems often fail to replicate the spatial architecture and immune complexity of native tumor microenvironments, thereby constraining their physiological relevance [78]. Compounding this issue is the lack of standardized culture protocols, which contributes to substantial inter-laboratory variability and undermines the reproducibility of experimental results [79]. Moreover, current models struggle to maintain appropriate stromal-to-epithelial cell ratios; the overgrowth of cancer-associated fibroblasts, for instance, can obscure tumor-specific characteristics and confound data interpretation. The genetic integrity of organoids also poses a concern, as extended *in vitro* expansion has been associated with clonal drift and the gradual loss of driver gene mutations, ultimately diminishing the representativeness of long-term cultures [19]. In addition, microbial contamination—particularly with fungi such as yeast and pseudohyphae—can interfere with organoid viability and formation during passaging procedures [80]. Finally, the efficiency of tumor tissue sampling remains a critical determinant of PDO establishment success, with variable yields depending on biopsy quality and cellular composition [81]. To overcome these limitations, future research must focus on refining organoid culture media [8,82], engineering more sophisticated co-culture systems that preserve cellular diversity and spatial structure [83,84], and implementing rigorous quality control strategies to maintain genomic fidelity over prolonged cultivation periods [85–87].

The integration of organoid technology with other advanced methods will open up new avenues for its applications. For instance, the integration of spatial omics technologies (e.g., Deterministic Barcoding in Tissue for Spatial Omics Sequencing [DBiT-seq]) enables simultaneous mapping of RNA and protein expression profiles in the tissue context, thereby providing a means to evaluate whether PDOs can accurately recapitulate the native spatial cellular organization and biomarker localization [26,28]. The integration of single-cell sequencing technology facilitates precise analysis of cellular heterogeneity and dynamic evolutionary patterns within organoids [22,88]. Circulating tumor cells (CTCs) derived from liquid biopsies and isolated from peripheral blood can serve as the basis for constructing three-dimensional organoid models, which are applicable for drug screening and disease modeling [89,90]. Endoscopic biopsy specimens have shown significant potential in establishing EAC organoids [86]. Tumor organoid-on-a-chip platforms, which combine PDOs with microfluidic chips, can precisely simulate physiological factors such as nutrient gradients, fluid shear stress, and immune cell perfusion. Meanwhile, microfluidic chip technology itself enables high-throughput simulation and real-time monitoring of the TME, providing a dynamic platform for studying tumor-immune interactions. These systems exhibit superior dynamic modeling capabilities and enhance the translational relevance of *in vitro* assays [25,27,91]. Furthermore, multi-omics approaches integrating single-cell RNA sequencing, spatial proteomics, and exome sequencing help dissect cellular heterogeneity and clonal dynamics within organoids, thereby uncovering drug resistance mechanisms and tumor evolution [13,17,30]. The incorporation of the air-liquid interface (ALI) and advanced co-culture systems containing immune cells, endothelial cells, and matched stromal components further enhances the ability of organoids to simulate interactions in the complex TME, such as lymphangiogenesis and crosstalk between CAFs and immune cells [5,19,21].

Moreover, the adoption of 3D bioprinting technology offers a new solution to improve the structural complexity of organoids and overcome the limitations of traditional extracellular matrices (e.g., Matrigel); its introduction may further enhance organoid function, making them more similar to *in vivo* tumor states [92–94]. Similarly, the use of synthetic hydrogels provides a new solution to improve structural complexity and address the limitations of traditional extracellular matrices like Matrigel. As alternatives to Matrigel, synthetic hydrogels show great promise and offer new strategies for cell repopulation and tissue regeneration in cancer therapy [93–95]. Positron emission microscopy (PEM) is an emerging tool that can be used for metabolic imaging, functional evaluation, and high-resolution detection of metabolic activity of tumor organoids in response to treatment, serving as a powerful tool for personalized therapy and drug screening [96].

**Figure 2:**
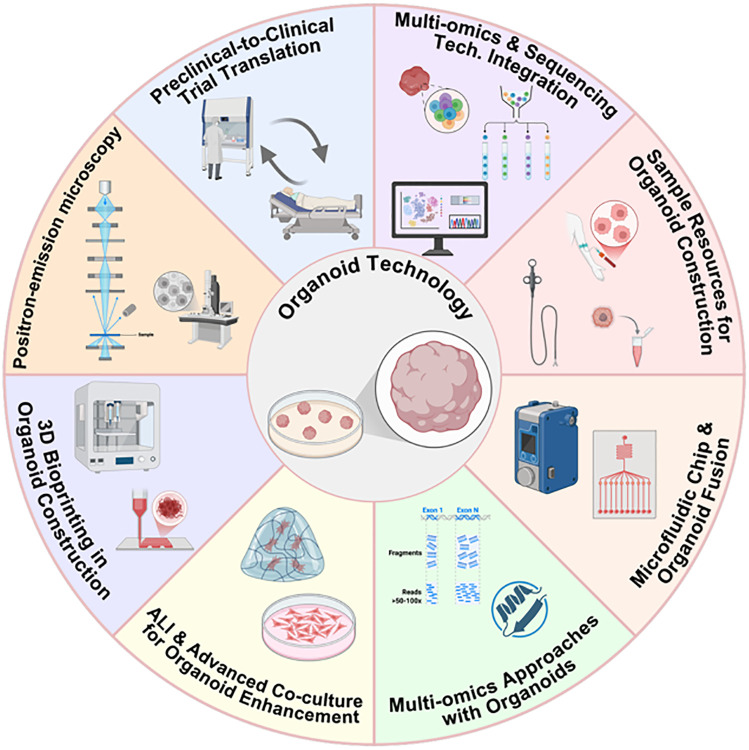
Schematic illustration of the integration of organoid technology with advanced methodologies (This figure was created using BioRender, available at https://biorender.com/; developed by BioRender Inc., Toronto, ON, Canada)

Beyond these technical integrations, a seamless “preclinical-to-clinical trial” model based on organoids is currently under exploration; notably, among the various areas summarized in Fig. 2, this translational model stands out as the most promising, as it directly addresses the core bottleneck of organoid technology by bridging preclinical research and clinical practice. This approach directly translates organoid drug sensitivity results into clinical trial design, potentially shortening drug development timelines and improving treatment success rates. Currently, two relevant clinical trials targeting head and neck cancer and its subtypes have been registered on ClinicalTrials.gov to advance this translational application: 1) NCT06686342 (titled “PDO Based Drug Sensitive Test in R/M HNSCC”), a prospective multicenter observational study that aims to evaluate the consistency of drug efficacy between clinical systemic treatment and patient-derived organoid (PDO)-based drug sensitivity testing in patients with recurrent/metastatic (R/M) HNSCC, with the design of multicenter observation to increase the generalizability and reliability of research conclusions; 2) NCT06482086 (titled “Efficacy of Organoid-Based Drug Screening to Guide Treatment for Locally Advanced Thyroid Cancer”), which focuses on exploring the potential advantages of anti-cancer therapy implemented based on organoid-derived drug sensitivity testing, enrolling individuals with locally advanced thyroid cancer who have undergone conventional therapy in the past or have unresectable tumors. It is expected to accelerate the development of therapeutic strategies, shorten the drug development cycle, and concurrently improve both the accuracy of response prediction and the success rate of treatments [19,25,97]. These innovations—encompassing the aforementioned technical integrations and the clinical translation model (summarized in Fig. 2)—reflect the evolving trend of integration between organoid platforms and advanced methods, with an overview of these technical and translational integrations also presented in Fig. 2.

Head and neck cancer organoids hold notable strengths, such as recapitulating tumor heterogeneity, enabling high-throughput drug screening, and being derivable from minimally invasive samples (e.g., endoscopic biopsies). However, they face limitations including incomplete TME simulation, reliance on animal-derived Matrigel, and lack of standardized protocols. Future efforts should focus on addressing these issues: using co-culture or organoid-on-chip systems to optimize TME mimicry, developing synthetic hydrogels as Matrigel alternatives, and establishing consensus protocols for different subtypes.

## 6 Conclusion

In conclusion, as technological advancements continue to progress, organoids will play an increasingly vital role in precision medicine for head and neck cancer. By integrating spatial biology validation, organoid-on-chip systems, multi-omics analytics, and standardized co-culture strategies—along with long-term genomic fidelity assurance and clinical trial pipelines—organoid models are positioned to evolve from descriptive systems to predictive platforms, thereby offering renewed hope for patients.

## Data Availability

Not applicable.

## Abbreviation

**Abbreviation**: **Full Name**
2D: Two-dimensional
3D: Three-dimensional
5-FU: 5-fluorouracil
AKT: Protein kinase B
AMPK: AMP-Activated Protein Kinase
ATC: Anaplastic thyroid cancer
AUC: Area under the curve
BMP: Bone morphogenetic protein
BRD: Bromodomain-Containing Protein
CAF: Cancer-associated fibroblast
CAR-NK: Chimeric antigen receptor-natural killer
CCL: Chemokine (C-C motif) ligand
CES1: Carboxylesterase 1
COSMIC: Catalogue Of Somatic Mutations In Cancer
CRISPR: Clustered regularly interspaced short palindromic repeats
cMET: Mesenchymal-Epithelial Transition Factor
EC: Esophageal Cancer/Epidermoid Carcinoma (subtype)
EC50: Half-maximal effective concentration
EAC: Esophageal adenocarcinoma
EGFR: Epidermal Growth Factor Receptor
EMT: Epithelial-mesenchymal transition
ESCC: Esophageal Squamous Cell Carcinoma
FGF: Fibroblast Growth Factor
FGFR: Fibroblast Growth Factor Receptor
HGF: Hepatocyte growth factor
HPV: Human papillomavirus
HNSCC: Head and neck squamous cell carcinoma
IC50: Half-maximal inhibitory concentration
LDH: Lactate dehydrogenase
LET: Linear energy transfer
MAPK: Mitogen-activated protein kinase
miRNA: microRNA
MSE C: Mixed squamous cell, epidermoid carcinoma (subtype)
NADH/NAD^+^: Nicotinamide Adenine Dinucleotide (Reduced Form/Oxidized Form)
NGS: Next-Generation Sequencing
NIS: Sodium-iodide symporter
NNMT-LOX-FAK: Nicotinamide N-Methyltransferase—Lysyl Oxidase—Focal Adhesion Kinase
OSCC: Oral squamous cell carcinoma
PDX: Patient-derived xenograft
PFCA: Perfluoroalkyl carboxylate
PG: Prostaglandin
PI3K: Phosphatidylinositol 3-kinase
PRMT5: Protein arginine methyltransferase 5
PTC: Papillary thyroid carcinoma
PTP: Protein Tyrosine Phosphatase
ROCK: Rho-Associated Protein Kinase
RSPO: R-spondin
RNAi: RNA Interference
ROS: Reactive Oxygen Species
SC: Squamous cell carcinoma (subtype)
SNP: Single-nucleotide polymorphism
SSC: Salivary Gland Secretory Carcinoma
Tg: Thyroglobulin
TGF: Transforming Growth Factor
TME: Tumor microenvironment
TSHR: Thyroid-Stimulating Hormone Receptor
Treg: Regulatory T Cells
WES: Whole-Exome Sequencing
Wnt3a: Wingless/Integrated 3a
